# Scopolamine-Morphine Narcosis in Childbirth

**Published:** 1919-02-22

**Authors:** 


### THE RELATIVE VALUE OF TWILIGHT SLEEP.

### Scopolamine-Morphine Narcosis in Childbirth.

A few years after its introduction by Gauss in 1906, this form of treatment was heralded by the lay Press with such acclamation that, even to-day, a large percentage of the general public take an extraordinarily optimistic view of its application, and in some quarters withholding of the method is still viewed as a typical instance of supposed lack of enterprise and initiative on the part of the medical profession of this country. There could not be a grosser misrepresentation of fact. Throughout many journalistic discussions and attacks, obviously conducted with but very superficial knowledge of the subject, a tendency has arisen to idealise, to take extreme standpoints, and especially to cite misleading instances, exceptional in either their success or their disaster. The matter was not realised as one upon which expert obstetricians might disagree, but rather considered as an example of a semi-secret method, absolutely safe and efficient in certain practised hands on the Continent, but which was unfortunately barred to most of the women in Great Britain through expense or professional obtuseness.

But nothing could be more misleading to the potential mother who dreams with varying dread of her approaching trials. Although we do not know the exact extent to which these drugs are in use by general practitioners throughout the country, yet it is certain that such means have been thoroughly tested and pronounced upon by our greatest experts, and a medical man once in possession of their results can readily explain to a patient the true perspective of the situation. His own judgment must here, as on so many occasions, decide what is to be suggested; she must make a guided choice and it is with a view to aiding a such joint conclusion that these few notes are penned.

### An Outline of the Method.

It is essential to understand exactly what is meant by "twilight sleep." It is neither analgesia nor anæsthesia, but a condition of amnesia. Sensation remains present though in diminished degree; the patient feels the pains of the uterine contractions; frequently she moans and in other ways shows that she is suffering, but these painful stimuli are not recorded in the memory cells of the brain, for after the influence of the drug is removed, she will maintain that she never experienced them.

The first effect of scopolamine in correct dosage is a pronounced weariness associated with muscular relaxation, and this stage is sometimes followed by one of motor-restlessness, but soon the patient passes into a sleep from which she is awakened by each sucessive pain. At this point the pains are clearly perceived both objectively and subjectively, but the amount of suffering they cause is in most cases diminished. In the majority of patients a further phase is then produced, and while they are under the influence of the drug sense impressions, although perceived, are no longer registered. Perception is not abolished, but "apperception"—the power of storing the perceptions—is lost.

It must be remembered, therefore, that precautions for the reduction of sensory impulses coming from outside are, in these cases, a particularly necessary provision. The labour-room must be kept absolutely quiet, darkened, and with some non-sounding material applied as a floor covering. The patient's ears should be plugged with cotton-wool and her eyes shaded, while all visits and examinations are conducted with the least possible disturbance. A nurse specially experienced in the treatment must be in constant attendance and professional aid must at all times be within the easiest reach; in other words, local conditions of an ideal standard are essential. Also it is clear that no standard dosage can be adopted since patients vary greatly in their reaction to the drug; if the amount be too small, amnesia will be incomplete, and there may remain "islands of memory" from which the woman can reconstruct the whole picture; if it be too great the most dangerous poisonous effects are produced in both mother and child.

### Compared with the Ideal.

Perhaps the easiest way to review the efficiency of the method is to draw a brief comparison with the properties of an ideal narcotic for spontaneous child-birth as enunciated by Gauss in his original paper. Let us take them in his sequence.

1. *There must be a marked reduction of pain.*

This object is undoubtedly achieved in all but the most unsuccessful cases.

2. *It must be free from unpleasant after-effects, or if such effects are inseparable from the action of the drug, they must be harmless to the patient.*

Here again, provided the question refers to the mother alone, it is hard to find fault. When dealing with labour, a process fraught with so much coincident unpleasantness for the woman, it is difficult to ascribe any of the known after-effects to the drugs themselves. After labour most patients sleep for a satisfactory period, while the puerperium and stage of strength recovery do not appear to be at all adversely affected. Also 75 per cent. of them are said to be able to nurse their children, a very good proportion under modern conditions, suggesting that lactation comes under no unfavourable influence.

3. *The normal progress of labour must not be interfered with during the dilatation of the cervix, the birth of the child, the separation and expulsion of the placenta or the arrest of hæmorrhage.*

In all cases there seems to be a fairly constant diminution in the strength and frequency of the contraction after the first dose, while later they tend to recover in spite of further scopolamine injections. Nevertheless, the fact makes a strong contra-indication for the method in primary uterine inertia. Also the frequent absence of voluntary

**The Relative Value of Twilight Sleep**—(*continued*).

bearing-down efforts tends to delay matters considerably. Of the second stage, one is justified in saying that in nearly all cases it is distinctly prolonged, especially in primiparæ and due to the above-mentioned causes or their complications. One direct result of this is a markedly increased forceps rate, not only from the normal cases which fail to deliver themselves spontaneously, but also the common non-rotation of occipito-posterior positions in the absence of effort by the patient. And, lastly, of the placenta; the third stage is sometimes quite normal but often prolonged and somewhat unsatisfactory.

4. *The child must not be injuriously affected in intra-uterine life; after delivery there must be no interference with the initiation of its physiological functions or with its later development.*

One cannot find any extensive observation on the foetal condition, but most records of the heart-beat show little departure from those usual in protracted labours, and practically all the still-births occurring can be readily accounted for by circumstances other than the drug effects. Yet, apart from forceps cases, a large number are born in a condition known as olignopnoea and this is stated as undoubtedly due to the combined action of the morphia and scopolamine. The child usually gives a cry as it is born but then makes no further attempt at respiration. It becomes first blue, then pale in colour; the heart-beat falls in some cases to 80 per minute, and the action often becomes irregular. Alarming enough at first but if the state be not considered as white asphyxia, and the child merely covered and kept warm, in favourable cases in ten to twenty minutes slow and shallow respirations commence, become deeper, and in a short time the normal rhythm is established.

### Percentage of Success.

Such, then, is a rough sketch of the process, and it is unnecessary to point out its advantages for the patient's own sensations if fortune favours her with complete amnesia. Given only partial success, almost the whole of these are at once lost. In fact a great many of the "twilight sleep" cases only obtain any approximation to loss of memory by the frequent custom that chloroform is employed to mask the pains at the birth of the head. Supporters of the method give as their best figure for complete amnesia 80 per cent., but this includes the chloroform cases. Without this all-important adjunct the percentage falls to 50.

There is a high proportion of failures, partial or complete, of all that the woman herself aims to gain, namely, an absence of memory for the event, and it is imperative that she should realise that a great deal is being staked on an uncertain chance. However normal her case at the start, she adds undoubted risk of her own life and a greater of that of her child.

### Demands upon Attendants.

The demands made upon the doctor himself are tremendous, the patient must be closely watched and not left alone for a minute whilst under the influence of the drug. She has to be seen at intervals of an hour over periods sometimes extending the whole clock round; the attendant must be prepared to remain with her during a second stage of four or five hours and even then may be called upon to wait another two or three when the birth is over. Such conditions are possible in a hospital where relays of skilled assistants are available, but almost out of the question in the average private house and much more training and experience than the ordinary maternity nurse can command are needed. It cannot be too strongly impressed that with prolongation of labour, the possibilities of introducing abnormalities into otherwise simple births are tremendous. The drugs, once injected, are largely out of the doctor's control—there being little chance of undoing any harm that may have been done—and they come under a very different category to the ordinary mask of the anæsthetist.

### General Adoption of System Condemned.

**It would be most unfortunate if the impression persist that there is not a considerable interference with the normal course of labour in all instances or that its skilled administration is entirely without risk, for this much the most enthusiastic specialist will at once admit, although he may prove that in his own or other competent hands the danger is reduced to a minimum.**

**It is obvious from all reliable records in this country that the treatment tends to produce uterine inertia, and to raise the forceps rate; it is impossible to raise the forceps rate without increasing the morbidity rate and the danger to the mother and the child.**

**This fact has been proved beyond question by various observers, and in itself is sufficient condemnation of the general adoption of so-called "twilight sleep" when we are at a point in the nation's history where infant welfare and the care of a coming generation are so universally important.**

